# Counseling for Physical Activity in Adults during the COVID-19 Pandemic: A Scope Review

**DOI:** 10.3390/ijerph19148687

**Published:** 2022-07-17

**Authors:** Letícia Gonçalves, Mikael Seabra Moraes, Diego Augusto Santos Silva

**Affiliations:** Research Center in Kinanthropometry and Human Performance, Sports Center, Department of Physical Education, Campus—Trindade, Federal University of Santa Catarinan, n. 476, Florianópolis 88040-900, Santa Catarina, Brazil; leticia.g.2008@hotmail.com (L.G.); moraesmikael@gmail.com (M.S.M.)

**Keywords:** health promotion, adult health, public health, motor activity

## Abstract

Objective: The aim of this scope review was to map the available scientific evidence on physical activity counseling for adults during the COVID-19 pandemic. Methods: The search was performed in PubMed, Web of Science, Scopus, SPORTDiscus, LILACS, SciELO, and CINAHL databases. Studies that described the population of adults over 18 years of age that used physical activity counseling during the COVID-19 pandemic context were selected. Data extracted were author, study location, sample, age group, sex, population characteristics, design, means used for intervention, time of intervention, professionals involved, and intervention or counseling strategy. Results: Physical activity counseling interventions were aimed at participants with insufficient levels of physical activity or with comorbidities; counseling was carried out in the online format; by health professionals, in the highest proportion of coaches, physicians, researchers, and nutritionists; through educational contents regarding the practice of physical activity; and using the transtheoretical model of behavior change as a reference method. Conclusions: The results of this review can provide tools for health professionals to assist in the process of coping with physical inactivity.

## 1. Introduction

Physical activity is broadly defined as any bodily activity that improves or maintains general health and fitness [1,2]. Scientific evidence has reported that regular physical activity significantly reduces the risk of all-cause mortality [3,4], chronic noncommunicable diseases [5], and types of cancers [5,6,7]. In the context of the COVID-19 pandemic, encouraging physical activity has become a global public health priority because increasing physical activity levels during this period resulted in reduced harm to physical health and improved immune function [8,9,10] and reduced risk of systemic inflammation, which resulted in lower chances of mortality from COVID-19 [10]. In addition, increased physical activity levels during the COVID-19 pandemic improve mental health [11]. A study showed that satisfactory levels of physical activity were associated with greater well-being, quality of life, and lower depressive symptoms such as anxiety and stress, regardless of age, during the first year of the COVID-19 pandemic [11].

Countries have presented alarming data on physical inactivity, with prevalence increasing with age, and it is higher among females when compared to males [12,13,14]. In addition, during the pandemic caused by the SARS-CoV-2 virus (severe acute respiratory syndrome-coronavirus), physical activity levels have declined [15,16] due to COVID-19 restrictions such as lockdowns, quarantine measures, and social distancing. Because of this, meeting physical activity guidelines posed a significant challenge [17,18,19]. Studies on the impact of physical inactivity caused by COVID-19 are still being explored; however, they have identified that reduced levels of physical activity may lead to greater chances of negative outcomes related to physical and mental health [20]. Other studies identified that adults infected with COVID-19 who did not meet the physical activity guidelines were more likely to be hospitalized and died when compared to those who met the physical activity guidelines [10]. In this period, it was found that fragile populations, which had multiple comorbidities such as diabetes, hypertension, and cardiovascular diseases, are more exposed to the severe clinical condition of SARS-CoV-2 [21]. This way, maintenance of physical activity during the pandemic context has been recommended [22], as it presents better control over these comorbidities [21], immunological benefits [9], and other health-related positive effects [15].

Physical activity counseling can be defined as advice and discussions about the practice of physical activity between health professionals and patients [23]; way of acting that involves listening, understanding people, and supporting them to plan and make more favorable decisions [24]; and general and structured guidelines aimed at encouraging the practice of physical activity in different domains [25]. Counseling interventions can include goal setting, self-monitoring, feedback, incentives, and problem solving [26]. Many studies have shown that counseling interventions can be effective strategies for promoting physical activity, have low costs [27], are replicable, and sustain physical activity for more than 12 months [23]. However, interventions that help individuals to engage in health-related beneficial behaviors, including physical activity, during the pandemic context are limited [22].

Thus, knowing evidence about the characteristics of counseling interventions that help reduce physical inactivity in adults, potentiated by the COVID-19 pandemic, is necessary. To our knowledge, currently no review presented this information. Thus, the aim of this study was to map the scientific evidence on physical activity counseling for adults during the COVID-19 pandemic.

## 2. Methods

This is a scope review whose guiding question was: What is the scientific evidence on physical activity counseling for adults during the COVID-19 pandemic? The research protocol was registered in the Open Science Framework (https://osf.io/e7jtv/, accessed on 6 December 2021), with writing of the text according to recommendations of the Preferred Reporting Items for Systematic reviews and Meta-Analyses extension for Scoping Reviews guide (PRISMA-ScR) [28] and the Joanna Briggs Institute (JBI) method [29]. This review followed six steps: identification of the research question; identification of relevant studies; selection of studies; data analysis; grouping, data synthesis, and presentation; and quality assessment of the risk of bias [29].

### 2.1. Study Eligibility Criteria

Inclusion criteria were: (a) original quantitative or qualitative articles with cross-sectional, longitudinal design, case-controls, cohort studies, interventions, or randomized clinical trials; (b) technical and governmental documents; (c) publications in English, Spanish, and Portuguese; and (d) all studies published until 3 December 2021. Exclusion criteria were: (a) narrative and integrative reviews, theoretical essays, conference abstracts; (b) articles not available in full in databases, and that all possibilities of accessing the texts have been exhausted, such as sending an email to the authors; and (c) articles that did not present data classifying population, concept, and context.

### 2.2. Selection of Evidence Sources

Two reviewers (LG and MSM) independently examined each database for potential articles. After extracting articles from databases, duplicate articles were excluded and then articles were excluded after reading titles and abstracts. Subsequently, the texts of selected articles were read in full for the selection of studies. Disagreements between the two reviewers were resolved by consensus meeting. If disagreements were not resolved, the opinion of a third reviewer (DASS) was consulted. The Rayyan software (Intelligent Systematic Review) was used to manage the studies found, whose functions allow the identification and exclusion of duplicate studies and division and organization of the results of each database, simultaneously and in a blind system. Selected articles underwent a final screening, after being read in full, and those that met the inclusion criteria were exported to the Zotero^®^ bibliographic manager version 5.0 (Roy Rosenzweig Center for History and New Media, Fairfax, VA, USA).

### 2.3. Search Strategies

Considering the inclusion criteria, a search strategy was developed for groups of descriptors inserted in the Medical Subject Headings (MeSH) platform, plus keywords selected by consensus in published scientific sources: (((adult[Text Word]) AND (Counseling[Text Word] OR Counselling[Text Word] OR Counseled[Text Word] OR Counselings[Text Word] OR Counsellings[Text Word] OR Counselled[Text Word] OR Counsels[Text Word] OR “Health promotion”[Text Word] OR “Health education”[Text Word])) AND (Exercise[Text Word] OR “Physical activity”[Text Word] OR “Motor activity”[Text Word])) AND (“COVID-19”[Text Word] OR “SARS-CoV-2”[Text Word] OR Coronavirus[Text Word]) (PubMed). This strategy was adapted according to the database used. The following databases were used: (1) PubMed via the National Library of Medicine (MEDLINE); (2) Web of Science; (3) Scopus; (4) SPORTDiscus via EBSCOhost; (5) LILACS via the Virtual Health Library; (6) Scientific Electronic Library Online (SciELO); and (7) Cumulative Index to Nursing and Allied Health Literature (CINAHL) via EBSCOhost. Descriptor groups were: (1) population (adults); (2) concept (physical activity counseling); and (3) context (COVID-19 pandemic). As additional resource, manual searches were performed in the references of selected articles. More details are described in Supplementary Document S1.

### 2.4. Risk of Bias Assessment

The risk of bias of included studies was independently assessed by two researchers (LG and MSM). For cases of disagreement between researchers, a third researcher was consulted through a consensus meeting (DASS). To assess the risk of bias, the tool proposed by the National Heart, Lung, and Blood Institute (NIH) [30] was used according to the type of study. For intervention studies with control group, the Quality Assessment Tool for Controlled Intervention studies tool was used and for intervention studies without control group, the Before-After (Pre-Post) Quality Assessment Tool was used. For each criterion evaluated, “no”, “yes”, “not reported”, and “not applicable” were assigned. The total score was obtained by adding the score of each question answered as “yes” (+1), while “no” and “not reported” were negatively counted (zero) and the criterion “not applicable” was excluded from the calculation [30].

## 3. Results

**Figure 1:**
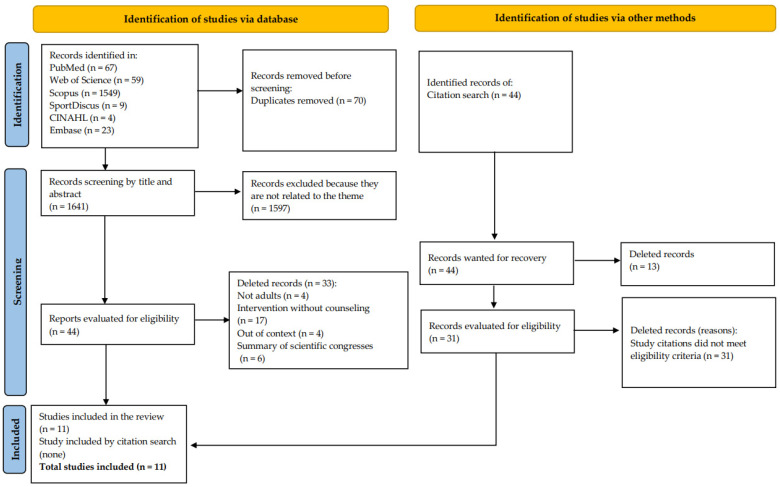
Flowchart of the studies selected via the database and via other methods.

A total of 1641 records, removing duplicates, were identified in this scope review. After reading titles and abstracts, 44 studies (2.7%) were considered eligible for full reading. Of this total, 31 were excluded because they did not meet the eligibility criteria, totaling 11 articles in the review (0.67%) via search in databases. No records were added after reading references (citation search); therefore, 11 articles were included for descriptive synthesis (Figure 1).

### 3.1. Characteristics of Records

All selected records were original articles (n = 11; 100%). Studies were conducted in seven countries (United States of America, United Kingdom, Netherlands, Spain, China, Taiwan, and Australia). The United States of America was the country with the highest number of records (n = 5; 45.4%).

### 3.2. General Sample and Population Characteristics

The sample size ranged from 7 [31] to 137 participants [32]. In addition to representing a majority in all samples, three articles were conducted specifically with female samples [14,33,34]. Heterogeneity was identified in relation to the sample characteristics, highlighting four samples composed of participants with insufficient levels of physical activity at the baseline [14,16,22,35], two samples with cancer survivors [32,34], and two with type II diabetes patients [36,37]. Regarding the objectives of the studies, six articles directed interventions to physical activity [31,32,33,34,35,38], two studies were focused on behavioral change [36,37], two studies investigated a whole lifestyle [16,22], and one study directed actions towards physical activity and behavioral change [14]. Regarding the research design, the selected studies were classified as randomized controlled trials (n = 4), intervention studies (n = 4), non-randomized controlled trials (n = 1), longitudinal (n = 1), and quasi-experimental (n = 1). Regarding the data analysis used, the studies were designated as quantitative (n = 8) or qualitative (n = 3) (Table 1).

### 3.3. Characteristics of PA Counseling during the COVID-19 Pandemic

Table 2 shows that the means used to carry out intervention were online platforms (n = 10; 90.9%) [14,16,22,32,33,34,35,37,38], including Facebook, YouTube, Google Classroom, Email, Zoom, WebEx; online applications [31,37]; telephone contact (n = 3; 27.3%) [32,33,36]; and face-to-face care (n = 2; 18.2%) [16,36]. Some studies reported more than one form of intervention, such as telephone contact and online platforms [32,33], telephone contact and face-to-face care [36], online platform and face-to-face care [16], and online platforms and online application [37]. High heterogeneity was observed in relation to the intervention time, ranging from 4 weeks (one month) [34] to 24 weeks (six months) [38].

Interventions were delivered by coaches (n = 3; 27.3%) [14,16,36]; physicians (n = 2; 18.2%) [32,38]; researchers or project managers (n = 2; 18.2%) [37]; nutritionists (n = 2; 18.2%) [16,22]; exercise physiologists (n = 1; 9.1%) [22]; nurses (n = 1; 9.1%) [32]; physical therapists (n = 1; 9.1%) [32]; professional caregivers (n = 1; 9.1%) [31]; a master’s student in public health (n = 1; 9.1%) [36]; and trained interviewers (n = 1; 9.1%) [33] (Table 1). Among professionals who performed the intervention, health professionals stood out (i.e., professionals working in the field of health sciences) [39] (n = 5; 45.4%) [22,31,32,38].

Most interventions provided educational contents regarding the practice of physical activity (n = 8; 72.7%) [14,16,22,31,32,33,34,35]. Contents were related to physical activity or exercise recommendations (n = 4; 36.3%) [16,22,32,35], benefits of physical activity (n = 1; 9.1%) [32], physical activity guidelines (n = 3; 27.3%) [31,32,35], negative effects of sedentary behavior and/or reduction of sedentary behavior (n = 3; 27.3%) [14,22,35], and general practices of healthy behaviors (n = 2; 18.2%) [33,34].

It was identified that five records used the transtheoretical model (behavioral change) as reference method to provide physical activity counseling [22,31,32,36,37]; five articles did not use reference methods [11,13,28,29,33]; and one used the self-determination theory method [35]. Concerning the applied methods, the majority of interventions used goal setting (n = 4; 36.3%) [32,33,36,37], followed by motivational interviewing (n = 4; 36.3%) [16,31,32,37], communication (n = 1; 9.1%) [31], competences (n = 1; 9.1%) [33], autonomy (n = 1; 9.1%) [33], barriers (n = 2; 18.2%) [32,37], feedback (n = 1; 9.1%) [37], practical recommendations (n = 1; 9.1%) [16], self-monitoring [32,37], social support (n = 1; 9.1%) [37], brief reminders to perform physical activity (n = 1; 9.1%) [38], and messages of congratulations to those who practice physical activities (n = 1; 9.1%) [38].

The results identified after the counseling intervention indicated that six studies increased the participants’ physical activity levels (i.e., indicators of physical activity and/or exercise) [14,16,22,33,35,36], and one study reported that there was no increase in physical activity levels [32]. None of the studies have identified that the intervention decreased the physical activity levels (or in the indicators of exercise and/or sport) of the participants. For four studies [31,34,37,38], it was not possible to identify the effect of the intervention on physical activity levels or other variables because they proposed to analyze the participants’ feedback on the interventions performed. 

### 3.4. Risk of Bias Assessment

The risk of bias/methodological quality was analyzed considering the general score of studies included in the review (11 articles) (Table 3). Among controlled intervention studies and pre- and post-intervention studies with a control group and without a control group, the study by Mcdonough et al. (2021) obtained the highest score, while the study by Jiwani et al. (2021) obtained the lowest score (Table 3).

## 4. Discussion

The main findings of this review were: (1) most physical activity counseling interventions during the COVID-19 pandemic were performed for participants with insufficient levels of physical activity or comorbidities; (2) most records on physical activity counseling interventions were provided by researchers, in the online format; (3) physical activity counseling interventions were performed by health professionals; (4) the instruments most used in counseling interventions were educational contents regarding the practice of physical activity; (5) most records used the transtheoretical model of behavior change as reference method; and (6) physical activity counseling interventions increased participants’ physical activity levels.

Included records showed that most participants who received physical activity counseling intervention had insufficient levels of physical activity or comorbidities. The physical activity counseling carried out during the COVID-19 pandemic represents an opportunity to address the issue and support the change in behavior and healthy habits, given that physical inactivity can bring negative health outcomes, associated with noncommunicable chronic diseases recognized as factors contributing to compromising clinical conditions and deaths in the pandemic scenario [40]. Additionally, physical activity counseling received by people with insufficient levels of physical activity or those with comorbidities supposedly has a positive effect, demonstrating that professionals recognize evidence of physical activity as a relevant factor in the treatment of these conditions [27]. However, counseling should also include individuals without comorbidities, regardless of sex or age [27], given that, in addition to the benefits of the practice of physical activity [41], it can also contribute to mental health related to the pandemic scenario [42].

The results of the present study indicated that physical activity counseling interventions for adults during the COVID-19 pandemic were carried out in the online format [14,16,22,32,33,34,35,37,38]. A possible justification would be the fact that the protective measures imposed by the pandemic to prevent the spread of the virus (for example, social distancing, isolation, and the closing of establishments) caused most participants to stay at home [31]. Thus, this may have led to increased accessibility and greater relevance in the use of digital platforms by various population groups, making them viable in the conduct of health interventions [43]. In addition, with the use of digital platforms, direct contact with other individuals is avoided, contributing to the containment of the COVID-19 pandemic [44], also presenting the following advantages: information and interactions in real time without the need or displacement of professionals or participants and low cost [43]. Thus, digital platforms are viable and strategic means to engage participants in physical activity and support health-related behavior change [45]. However, it should be considered that the adoption of these technologies can be fragmented [46], especially in low and middle-income countries, since individuals do not have equitable access to digital technologies, either due to lack of adequate devices or internet access [47,48].

Physical activity counseling interventions were carried out by health professionals, mostly coaches, physicians, researchers, and nutritionists. Other review studies with similar themes have identified different results, reporting that physicians had higher proportion of counseling interventions [24,27]. However, the findings in the present review suggest expanding the scope of action and practice of health professionals, since counseling interventions are not the responsibility of a single professional category but of a multidisciplinary team [49]. Additionally, any professional should be able to implement educational and counseling actions, adding experiences for the development of techniques that synthesize better information in different fields of activity [50].

The present study pointed out that the instruments most used in counseling interventions were educational contents regarding the practice of physical activity, including recommendations for physical activity or exercises [16,22,32,35], benefits of PA practice [32], and PA guidelines [31,35]; negative effects or reduction of sedentary behavior [14,22,35]; and general practices of healthy behaviors [33,34]. The findings suggest that since the lack of standardization of contents covered and the knowledge about physical activity guidance and advice as education strategy are still scarce [51], receiving common information (for example, benefits of physical activity and harmful effects of sedentary behavior) can raise awareness and influence most participants to engage in physical activities [52]. However, when considering the barriers imposed during the pandemic period, it is necessary to verify which content will be implemented according to the reality and needs of participants [52]. Therefore, the use of health education strategies can enable changes in professional practices and encourage the search for healthy behaviors [51].

The records included indicated that the most discussed reference method for counseling was the transtheoretical model (behavioral change) [22,31,32,36,37]. These findings can be explained by the fact that such behavior change model is a structured and widely recognized approach to counseling to facilitate healthy behaviors [53]. Additionally, changes are gradually observed through a cyclical process of stages [31], causing participants to set goals, face barriers, and build interrelationships with professionals, expanding possibilities to initiate or increase levels of physical activity [53]. Furthermore, it allows researchers to perform a quantifiable assessment of the behavior change stage in which the individual is in [53]. However, it should be explored during the pandemic period, integrating effective, viable, and acceptable solutions (e.g., performing physical activity safely, reflective exercises), especially for economically-disadvantaged participants.

The results identified in the present review showed that most counseling interventions increased participants’ physical activity levels [14,16,22,33,35,36]. In agreement with the results of the present study, other non-pandemic review studies identified that physical activity counseling interventions showed promising results in increasing physical activity levels in the short term [54,55], but evidence on the effectiveness of long-term interventions has not yet been explored. In this way, the findings of the present study give the direction that counseling interventions for physical activity in the pandemic period can be good strategies for people with insufficient physical activity levels [56], because it can help in the systematic performance of physical activity and reduce health problems [40,57]. In addition, increasing physical activity levels can help reduce the worsening of clinical conditions caused by COVID-19 [5,17,58,59,60,61]. Regarding the feasibility and acceptability of interventions, four studies [22,34,37,38] investigated this issue and reported that interventions were feasible, acceptable, and effective for improving functional fitness, preventing some of the consequences of physical inactivity and social isolation associated with the pandemic, increasing knowledge of health behaviors, and meeting important needs related to healthy living. 

The risk of bias/methodological quality was analyzed considering the overall scores of included studies. Some of the items included by the analysis instrument [30], evaluated in this review, contributed to the reduction of scores attributed to controlled intervention studies such as blinding for intervention evaluation and adherence to intervention protocols for each treatment group and the design of pre- and post-intervention studies without control group, sufficient sample size, and loss to follow-up accounted for in the analysis. Considering that the results of counseling interventions can impact both the reduction of physical inactivity [27] as well as the service provided to the population, a better analysis of the design and conduction of studies is justified, so that the evaluated outcomes are not submitted to comparison bias, providing knowledge of the scope and effectiveness of interventions.

This scope review mapped scientific evidence on physical activity counseling for adults during the COVID-19 pandemic. Common axes regarding types, methods, and means of carrying out interventions during the COVID-19 pandemic were reported. As study limitation, the search in the gray literature may not have been sufficient to cover the related content on the subject, especially because theses or dissertations were not included. However, eight databases were searched, in addition to the reading of references to increase sensitivity in the search for articles. Another limitation of this review was the selection of studies to obtain the information of interest. This is because we included studies in which the objective was to increase physical activity levels due to pandemic-related effects and studies which presented interventions carried out during the COVID-19 pandemic. However, it is noteworthy that all the selected articles had deleterious effects related to the pandemic context in the conduct of interventions or in the results, making them relevant for the purpose of this review. Although most of the studies identified in the present review showed a positive association with the investigated outcome, further reviews should be explored, providing secure evidence regarding the efficacy, feasibility, and acceptability of physical activity counseling interventions during the COVID-19 pandemic. 

Among practical implications, it could be mentioned that technological resources (online platforms, telephone contact, and applications) are strong allies to promote physical activity counseling interventions during the COVID-19 pandemic. In addition, physical activity counseling interventions are necessary for healthy subjects, or for those with comorbidities, regardless of sex or age, since physical activity can be beneficial to most participants, both for physical and mental health, especially during the pandemic period. It is important to highlight that counseling should be feasible and implemented through safe, viable, and effective strategies, considering the reality and needs of participants.

## 5. Conclusions

According to the findings of this review, interventions on physical activity counseling during the COVID-19 pandemic were provided by health professionals through technological resources, based on educational contents and behavioral strategies, and resulted in increases in participants’ physical activity levels. The importance of new records on the subject, capable of intervening during pandemic restrictions, is highlighted. The results of this review allow health professionals to assist in the process of coping with physical inactivity through interventions, educational practices, and informational materials, contributing to the physical and mental health of participants. Furthermore, the results found in this review showed that despite the different intervention methods used, most interventions on physical activity counseling resulted in increased physical activity levels and this can be considered support for decision-making and health interventions. Future reviews should explore the feasibility and applicability of different physical activity counseling interventions in different contexts.

## Figures and Tables

**Table 1:** Descriptive characteristics of the scoping review studies on counseling for physical activity during the COVID-19 pandemic.

Author/Year	Local	Sample	Age Range	Sex	Characteristics of the Population	Objective	Study Design	Data Analysis
(Mckeon et al., 2021) [22]	Australia	11	>60 years	F and M	Insufficient levels of physical activity	To determine the feasibility and preliminary effectiveness of delivering an online group lifestyle intervention for older adults during the COVID-19 pandemic.	Intervention	Quantitative
(Mcdonough et al., 2021) [35]	United States	64	18 to 35 years	F and M	Insufficient levels of physical activity and Body mass index (BMI) ≥ 18.5 kg/m^2^	To examine the effects of a home-based, YouTube-delivered PA intervention grounded in self-determination theory on young adults’ free-living PA, sedentary behavior, and sleep quality.	Controlled and randomized clinical trials	Quantitative
(Egan et al., 2021) [31]	United Kingdom (Scotland)	7	>18 years	F and M	Health Professionals (Formal and informal caregivers)	To co-design and develop a novel mobile app to educate and support caregivers in the undertaking of regular physical activity at home during and beyond COVID-19 restrictions via integration of the transtheoretical model of behavior change and UK physical activity guidelines.	Longitudinal	Qualitative
(Johnson et al., 2021) [33]	United States	13	>65 years	F	BMI ≥ 18.5 kg/m^2^	To examine how a telehealth intervention changed activity profiles in older adults during the COVID-19 pandemic.	Intervention	Quantitative
(Van de Wiel et al., 2021a) [32]	The Netherlands	137	>50 years	F and M	Breast and prostate cancer survivors	To develop an Internet-based physical activity (PA) support program (IPAS) and evaluate the effectiveness and costs of IPAS alone (online only) or IPAS combined with physiotherapist telephone counselling (blended care), compared to a control group.	Controlled and randomized clinical trials	Quantitative
(García Pérez de Sevilla et al., 2021) [16]	Spain	24	>18 years	F and M	Insufficient levels of physical activity	To evaluate the adherence to a lifestyle intervention carried out in university employees during the COVID-19 pandemic and its impact on health-related quality of life (HrQoL).	Controlled and randomized clinical trials	Quantitative
(Lin et al., 2021) [36]	Taiwan	104	20 to 75 years	F and M	Type II diabetes patients	To explore the impact of diabetes self-management and HbA1c affected by the COVID-19 pandemic and the epidemic prevention work.	Quasi-experimental	Quantitative
(Sciamanna et al., 2021) [38]	United States	24	>60 years	F and M	Patients in Primary Health Care	To explore the feasibility and impact of a PCP-prescribed one-minute daily functional exercise program, consisting of 30 s each of bodyweight push-ups and squats, among 24 patients 60 years of age or older.	Intervention	Quantitative
(Zhang et al., 2021) [14]	China	70	18 to 30 years	F	Insufficient levels of physical activity	To assess the effectiveness of an online high-intensity interval training (HIIT) intervention and health education on the behaviors, mental health, and cognitive function of sedentary young females.	Controlled and randomized clinical trials	Quantitative
(Jiwani et al., 2021) [37]	United States	18	>65 years	F and M	Type II diabetes patients, BMI ≥ 25	To explore participants’ acceptability and experiences following a behavioral lifestyle intervention that integrated Fitbit in overweight/obese older adults with T2D amid the COVID-19 pandemic.	Intervention	Qualitative
(Robertson et al., 2022) [34]	United States	16	28 to 82 years	F	Completion of primary cancer treatment.	To develop and characterize the relevance and potential utility of electronically delivered acceptance- and mindfulness-based approaches to physical activity promotion for insufficiently active breast cancer survivors.	Non-randomized trial	Qualitative

F: Female; M: Male; kg/m^2^: kilograms per square meter; BMI: body mass index.

**Table 2:** Synthesis of key findings on physical activity counseling during COVID-19.

Author/Year	Means Used for Intervention	Intervention Time	Professionals Involved	Used Tools	Reference Method	Other Strategies	Intervention	Results
(Mckeon et al., 2021) [22]	Online platform (Facebook)	6 weeks	Exercise physiologist and nutritionist	Educational contents of physical activity; Recommendations for physical activity or exercise; Negative effects or reduction of sedentary behavior	Transtheoretical model	Did not describe	Distribution of weekly content on physical activity. Facilitators provide education and discussion on goal setting, balance training, reducing sedentary behavior, and diet (posts and video calls in groups); The content was shaped by defined behavior change techniques, such as social support, self-monitoring, identification of barriers, and feedback.	The response rate of the intervention group was 91.0%. The intervention showed evidence of effect for the investigated outcomes (psychological distress, quality of life, sedentary time, function, loneliness, walking time, and moderate and vigorous physical activity).
(Mcdonough et al., 2021) [35]	Online platform (YouTube)	12 weeks	Project manager	Educational contents of physical activity; Recommendations for physical activity or exercise; Physical activity guidelines; Negative effects or reduction of sedentary behavior	Theory of self-determination	Did not describe	**Intervention Group:** home-based physical activity, with distribution of videos with aerobic exercises, muscle strength, health education videos, and health education related to physical activity; **Control group:** only weekly health education videos, with physical activity content; The interventions were delivered via an online platform (YouTube), based on the theory of self-determination on the practice of free lifestyle activities by young people, sedentary behavior, and sleep quality.	The intervention showed a significant association for the intervention group, in the variables moderate and vigorous physical activity, sleep efficiency, frequency of physical activity for muscle strengthening, motivation related to physical activity, and coping with barriers to physical activity (*p* < 0.001–0.002).
(Egan et al., 2021) [31]	Online application (CAREFIT)	6 months	Multidisciplinary team—health professionals, caregivers, and health specialists	Educational contents of physical activity; Physical activity guidelines	Transtheoretical model	Motivation; Communication	Custom application development (CAREFIT) with approaches based on national physical activity guidelines and behavior change models. The application addressed physical activity content, elements of education, communication, tools, or motivation to perform regular physical activity.	Results were based on feedback from participants regarding physical activity orientation, type of physical activity, intensity, educational sections, and others. Thus, it was not possible to identify an effect on the increase in physical activity levels.
(Johnson et al., 2021) [33]	Online platform (Google Classroom) and telehealth (telephone contact)	6 weeks	Trained interviewers	Educational contents of physical activity; General practices of healthy behaviors	Did not use reference method	Definition of goals; Skills; Autonomy	Distribution of teaching materials related to the practice of healthy lifestyle behaviors. Facilitators work individually with participants over the phone to discuss the intervention and develop a personalized physical activity strategy, encouraging participation in light and moderate to vigorous physical activity was emphasized, sense of relationship, and competence. The definition of goals was also used to encourage participants to practice physical activities, autonomy, feelings, and the reinforcement of competence in physical activities.	After completion of the intervention, moderate to vigorous participation in physical activity increased by an estimated 2 min/day (CI: −21, 26) and 12 min/week (CI: −154, 180), but this trend was not statistically significant.
(Van de Wiel et al., 2021a) [32]	Online platform (Physical Activity Support Program) and telephone contact	6 weeks	Doctors, nurses, and physiotherapists	Educational contents of physical activity; Recommendations for physical activity or exercise; Benefits of physical activity; Physical activity guidelines	Transtheoretical model	Definition of goals; Motivation; barriers; self-monitoring	**Control group:** received a printed leaflet with guidelines and possible benefits of physical activity after cancer treatment. It also introduced physical activity guidelines and provided information on monitoring and intensity of physical activity; **Online group:** received access to the Online Physical Activity Support Program (IPAS), structured according to the transtheoretical model, using aspects of the theory of planned behavior and social cognitive theory. Participants were separated into stages of behavior change and, according to each stage, received information, images, and interactive attributions and videos of physical activity; **Blended group:** Received access to IPAS and phone calls for physical activity counseling. Subjects were asked to exercise on a stationary bike or treadmill and to establish goals and targets for intended behavior change.	The intervention showed no significant association with moderate and vigorous physical activity between the online group and the control group (*p* = 0.39), and between the blended care group and the control group (*p* = 0.75). Additionally, of the 1242 invited patients, 137 participated in the study (participation rate: 11.0%).
(García Pérez de Sevilla et al., 2021) [16]	Online platform and face-to-face service	18 weeks	Nutritionists and Fitness Trainers	Educational contents of physical activity; Recommendations for physical activity or exercise	Did not use reference method	Motivation; Practical recommendations	**Intervention group:** Educational intervention on healthy habits, with distribution of 12 weekly videos on motivation for change, exercise recommendations, and exercise strategies. Afterwards, nutritional intervention and physical activity were performed. Carried out recommendations on physical activity and nutrition to maintain acquired habits in the long term; **Control group:** routine activities without supervision.	The intervention group showed a significant association with quality of life in the time and group interaction, (*p* = 0.03) health responsibility (*p* = 0.02), physical activity (*p* = 0.02), and nutrition (*p* = 0.02), with a large effect size for these four variables. Sitting time was reduced by 2.5 h per day, with a moderate effect size.
(Lin et al., 2021) [36]	Telephone contact and face-to-face service	6 months	Trainer and Master’s in Public Health	Did not describe	Transtheoretical model	Goal setting	**Intervention group:** coaching sessions via telephone, all for six months, on top of shared diabetes care, which includes setting goals for behavior change; **Control group:** a face-to-face coaching and baseline measurement session at the baseline without having any coaching calls; Both intervention and control groups received diabetes health education and usual care.	Positive associations were found between the intervention, setting physical activity goals. and physical activity indicator (*p* = 0.007).
(Sciamanna et al., 2021) [38]	Online platform (email)	24 weeks	Doctor	Did not describe	Did not use reference method	Brief reminders for physical activity; congratulatory message	Patients were advised to perform squatting and bending exercises, lasting 60 to 90 s. Moreover, brief messages were made available via email to remind participants to perform the exercises, congratulations and memes (humorous images) if the participant entered data about the exercise performed on the day, frequency with which other patients had completed the exercises, the longest daily sequence, and comments that other patients provided.	The intervention response rate was 42.0% over 24 weeks. The intervention showed a significant association with maximal performance in the squat (*p* < 0.001) and flexion over time (*p* < 0.0001), suggesting that one-minute counseling was feasible, acceptable, and effective for improving physical fitness.
(Zhang et al., 2021) [14]	Online platform (Zoom)	9 weeks	Professional coaches	Educational contents of physical activity; Negative effects or reduction of sedentary behavior	Did not use reference method	Did not describe	**Intervention group:** 6-week high-intensity interval training (HIIT) and health education intervention; **Control Group:** 6-week health education (body self-cognition, exercise, physical inactivity and health, nutrition and diet, fat reduction, muscle gain and shaping, stretching, meditation, relaxation and rehabilitation, and emotion regulation) and risk of sedentary behavior; Both groups received the same lecture content.	The response rate of the intervention group was 100.0% and that of the control group was 78.0%. Positive associations were found between the intervention group and moderate to vigorous physical activity (*p* < 0.001), while the control group demonstrated a reduction in sedentary time (*p* = 0.03). In addition, levels of anxiety (*p* = 0.002) and stress (*p* = 0.001) were shown to significantly reduce in the intervention group over the six-week period.
(Jiwani et al., 2021) [37]	Online platform (WebEx) and online application (FitBit)	6 months	Researcher	Did not describe	Transtheoretical model	Definition of goals; Motivation; Barriers; Feedback; Self-monitoring; Social support	Behavioral lifestyle intervention (diet and physical activity) with integration of self-monitoring (FitBit app). The facilitator used a publicly available behavioral intervention guide for the study sessions, focusing on adherence to behaviors using motivational strategies. Each participant was given a weight loss and physical activity goal.	The response rate of the intervention group was 90.0%. The results showed good acceptance by the participants of the intervention and with the use of Fitbit technology for self-monitoring of diet and physical activity, high degree of acceptability to the program, and participant motivation to continue to track behavior using program strategies.
(Robertson et al., 2022) [34]	Online platform	4 to 8 weeks	Did not describe	Educational contents of physical activity; General practices of healthy behaviors	Did not use reference method	Did not describe	Acceptance and mindfulness-based intervention (living flexibly) was delivered electronically through survey, and consisted of narrated videos with visual aids, audio files, images, text and accompanying documents, and email and text messages presenting techniques for promoting physical activity.	The results were based on feedback from participants, demonstrating that the content of the intervention was acceptable and relevant to meet needs related to healthy living.

**Table 3:** Assessment of the methodological quality of the studies included in the scoping review.

**Controlled Intervention Studies ^£^**						
Criteria	(Mcdonough et al., 2021) [35]	(Van de Wiel et al., 2021a) [32]	(García Pérez de Sevilla et al., 2021) [16]	(Lin et al., 2021) [36]	(Zhang et al., 2021) [14]	(Jiwani et al., 2021) [37]
Described as rehearsal	Y	Y	Y	Y	Y	Y
Proper randomization	Y	Y	Y	Y	Y	N
Hidden treatment	Y	Y	N	Y	Y	N
Blinded participants and providers	Y	N	N	Y	Y	N
Blinded evaluators	Y	N	N	Y	N	N
Similar groups	Y	Y	Y	Y	Y	Y
Abandonment rate 20% or lower	Y	N	N	Y	Y	Y
Dropout rate by 15 percentage points or lower	Y	N	N	Y	N	Y
High adhesion	Y	N	N	N	Y	N
Similar interventions were avoided	Y	N	Y	Y	N	N
Valid outcome	Y	Y	Y	Y	Y	N
Enough sample	Y	N	N	Y	Y	N
Outcomes have been reported	Y	Y	Y	Y	Y	N
Participants analyzed in the original group	Y	Y	Y	Y	Y	N
Total score *	1.0	0.5	0.5	0.9	0.7	0.3
**Before-After (Pre-Post) Studies with No Control Group ^£^**						
Criteria	(Johnson et al., 2021) [33]	(Mckeon et al., 2021) [22]	(Sciamanna et al., 2021) [38]	(Egan et al., 2021) [31]	(Robertson et al., 2022) [34]	
Clear objective	Y	Y	Y	Y	Y	
Selection eligibility	Y	Y	Y	Y	Y	
Eligible participants	Y	Y	Y	Y	Y	
Enrollment criteria	Y	Y	Y	Y	Y	
Enough sample size	N	Y	N	N	Y	
Intervention described to the Population	Y	Y	Y	Y	Y	
Valid outcomes	Y	Y	Y	Y	Y	
Blinding of evaluators	NR	NR	Y	N	N	
Follow loss of 20% or less	Y	N	NR	NR	N	
Losses were accounted for in the analysis	N	Y	Y	N	N	
Statistics performed before and after the intervention	Y	Y	Y	N	N	
P-value and pre-post-post analysis	N	Y	Y	NR	Y	
Measures of interest before and after the intervention	-	-	-	-	-	
Group-level intervention and individual-level analysis to consider group effects	-	-	-	-	-	
Total score *	0.7	0.8	0.8	0.5	0.7	

Questions for analyzing the quality of the studies (Y: Yes; N: No; -: Not applicable; NR: Not reported; 0: No; 1: Yes. * To determine the total score, the equation was considered: total positive responses/total number of questions considered in the study); ^£^: Questions for each study type are available at https://www.nhlbi.nih.gov/health-topics/study-quality-assessment-tools (accessed on 1 January 2022).

## Supplementary Materials

The following supporting information can be downloaded at: www.mdpi.com/article/10.3390/ijerph19148687/s1, Document S1: Base Search Strategy.
