# Supplementary material for: Counseling for Physical Activity in Adults during the COVID-19 Pandemic: A Scope Review
Source: Int J Environ Res Public Health. 2022 Jul 17;19(14):8687. doi: 10.3390/ijerph19148687 (PMC9322393; doi:10.3390/ijerph19148687)
Supplement: Supplementary file 1 [file ijerph-19-08687-s001.zip › ijerph-1788131-supplementary.pdf]

Base Search Strategy

1 – PubMed: 03/12/2021.		
**Block**	**Descriptors**	**Articles**
1	*Adult*	***All fields***: 8.336.063 ***Text Word*** 5.939.595
1 + 2	*Counseling OR Counselling OR Counseled OR Counselings OR Counsellings OR Counselled OR Counsels OR "Health promotion" OR "Health education"*	***All fields***: 375.725 ***Text word***: 314.167
1 + 2 + 3	*Exercise OR "Physical activity" OR "Motor activity"*	***All fields***: 657.934 ***Text word***: 537.248
1 + 2 + 3 + 4	*"COVID -19" OR "SARS-CoV-2" OR Coronavirus*	***All fields***: 218.900 ***Text word***: 214.650
(((Adult) AND (Counseling OR Counselling OR Counseled OR Counselings OR Counsellings OR Counselled OR Counsels OR "Health promotion" OR "Health education")) AND (Exercise OR "Physical activity" OR "Motor activity")) AND ("COVID -19" OR "SARS-CoV-2" OR Coronavirus)<br><br>*Filters applied: Year:2019,2020,2021,2022, Female, Male, Age: Adult: 19+ years, Young Adult: 19-24 years, Adult: 19-44 years, Middle Aged + Aged: 45+ years, Middle Aged: 45-64 years, Aged: 65+ years, 80 and over: 80+ years.*		*67*

2 - Web of Science. Search performed on: 03/12/2021.		
**Block**	**Descriptors**	**Articles**
1	*Adult*	***All fields***: 1.774.891 ***Title:*** 504.712

1 + 2	*Counseling OR Counselling OR Counseled OR Counselings OR Counsellings OR Counselled OR Counsels OR "Health promotion" OR "Health education"*	***All fields***: 278.283 ***Title:*** 54.587
1 + 2 + 3	*Exercise OR "Physical activity" OR "Motor activity"*	***All fields***: 778.272 ***Title:*** 256.165
1 + 2 + 3 + 4	*"COVID -19" OR "SARS-CoV-2" OR Coronavirus*	***All fields***: 236.096 ***Title:*** 193.219
ALL=((Adult) AND (Counseling OR Counselling OR Counseled OR Counselings OR Counsellings OR Counselled OR Counsels OR "Health promotion" OR "Health education") AND (Exercise OR "Physical activity" OR "Motor activity") AND ("COVID -19" OR "SARS-CoV-2" OR Coronavirus))		59

3 – Scopus. Search performed on: 03/12/2021.		
**Block**	**Descriptors**	**Articles**
1	*Adult*	***All fields***: 12.269.177 ***Title/Abstract/ Keywords***: 8.753.595
1 + 2	*Counseling OR Counselling OR Counseled OR Counselings OR Counsellings OR Counselled OR Counsels OR "Health promotion" OR "Health education"*	***All fields***: 1.380.626 ***Title/Abstract/ Keywords***: 508.986
1 + 2 + 3	*Exercise OR "Physical activity" OR "Motor activity"*	***All fields***: 2.155.515 ***Title/Abstract/ Keywords***: 924.285
1 + 2 + 3 + 4	*"COVID -19" OR "SARS-CoV-2" OR Coronavirus*	***All fields***: 400.659 ***Title/Abstract/ Keywords***: 274.668

(ALL (adult) AND ALL (counseling OR counselling OR counseled OR counselings OR counsellings OR counselled OR counsels OR "Health promotion" OR "Health education") AND ALL (exercise OR "Physical activity" OR "Motor activity") AND ALL ("COVID -19" OR "SARS-CoV-2" OR coronavirus)) AND PUBYEAR > 2018 AND (LIMIT-TO (OA , "all")) AND (LIMIT-TO (PUBYEAR , 2022) OR LIMIT-TO (PUBYEAR , 2021) OR LIMIT-TO (PUBYEAR , 2020) OR LIMIT-TO (PUBYEAR , 2019)) AND (LIMIT-TO (DOCTYPE , "ar") OR LIMIT-TO (DOCTYPE , "no") OR LIMIT-TO (DOCTYPE , "ed") OR LIMIT-TO (DOCTYPE , "bk") OR LIMIT-TO (DOCTYPE , "cp") OR LIMIT-TO (DOCTYPE , "ch") OR LIMIT-TO (DOCTYPE , "sh") OR LIMIT-TO (DOCTYPE , "dp")) AND (LIMIT-TO (SUBJAREA , "MEDI") OR LIMIT-TO (SUBJAREA , "NURS") OR LIMIT-TO (SUBJAREA , "HEAL")) AND (LIMIT-TO (EXACTKEYWORD , "COVID-19") OR LIMIT-TO (EXACTKEYWORD , "Pandemic") OR LIMIT-TO (EXACTKEYWORD , "Physical Activity") OR LIMIT-TO (EXACTKEYWORD , "Exercise") OR LIMIT-TO (EXACTKEYWORD , "Public Health") OR LIMIT-TO (EXACTKEYWORD , "Health Promotion") OR LIMIT-TO (EXACTKEYWORD , "Lifestyle") OR LIMIT-TO (EXACTKEYWORD , "Quality Of Life") OR LIMIT-TO (EXACTKEYWORD , "Health Behavior") OR LIMIT-TO (EXACTKEYWORD , "Health Care Personnel") OR LIMIT-TO (EXACTKEYWORD , "Telehealth") OR LIMIT-TO (EXACTKEYWORD , "Behavior Change") OR LIMIT-TO (EXACTKEYWORD , "Randomized Controlled Trial") OR LIMIT-TO (EXACTKEYWORD , "Health") OR LIMIT-TO (EXACTKEYWORD , "Attitude To Health") OR LIMIT-TO (EXACTKEYWORD , "Clinical Article") OR LIMIT-TO (EXACTKEYWORD , "Health Care Delivery") OR LIMIT-TO (EXACTKEYWORD , "Randomized Controlled Trial (topic)") OR LIMIT-TO (EXACTKEYWORD , "Health Care")) View less<br><br>*Filters applied: Year:2019,2020,2021,2022, Subject área: “Medi”, “Nurs”, “Heal”, “Mult”,*<br>*Keyword:*	1.549

4 - SPORTDiscus via EBSCOhost. Search performed on: 03/12/2021.		
**Block**	**Descriptors**	**Articles**

1	*Adult*	***All fields***: 78.912 ***Text Word*** 209.181
1 + 2	*Counseling OR Counselling OR Counseled OR Counselings OR Counsellings OR Counselled OR Counsels OR "Health promotion" OR "Health education"*	***All fields***: 43.323 ***Text word***: 93.588
1 + 2 + 3	*Exercise OR "Physical activity" OR "Motor activity"*	***All fields***: 297.102 ***Text word***: 420.882
1 + 2 + 3 + 4	*"COVID -19" OR "SARS-CoV-2" OR Coronavirus*	***All fields***: 1.538 ***Text word***: 5.766
TX Adult AND (Counseling OR Counselling OR Counseled OR Counselings OR Counsellings OR Counselled OR Counsels OR "Health promotion" OR "Health education") AND (Exercise OR "Physical activity" OR "Motor activity") AND ("COVID -19" OR "SARS-CoV-2" OR Coronavirus)		09

5 - LILACS via Biblioteca Virtual em Saúde. Search performed on: 03/12/2021.		
**Block**	**Descriptors**	**Articles**
1	*Adult*	***Words***: 190.906 ***Title:*** 5.385 ***Abstract***: 15.082
1 + 2	*Counseling OR Counselling OR Counseled OR Counselings OR Counsellings OR Counselled OR Counsels OR "Health promotion" OR "Health education"*	***Words***: 18.858 ***Title:*** 628 ***Abstract***: 1.953

1 + 2 + 3	*Exercise OR "Physical activity" OR "Motor activity"*	***Words:*** 17.679<br>***Title:*** 3.676<br>***Abstract*:** 8.041
1 + 2 + 3 + 4	*"COVID -19" OR "SARS-CoV-2" OR Coronavirus*	***Words:*** 7.705<br>***Title:*** 948<br>***Abstract*:** 2.119
((Adult) AND (Counseling OR Counselling OR Counseled OR Counselings OR Counsellings OR Counselled OR Counsels OR "Health promotion" OR "Health education") AND (Exercise OR "Physical activity" OR "Motor activity") AND ("COVID -19" OR "SARS-CoV-2" OR Coronavirus)) [Palavras].		*00*
1	*Adulto*	***Words:*** 205.142<br>***Title:*** 3.618<br>***Abstract***: 6.691
1 + 2	Aconselhamento *OR Consejo OR* Conselho *OR Asesoramiento OR* Aconselhou *OR "*Promoção da saúde*" OR "Promoción de la salud" OR "*Educação em saúde*" OR "Educación para la salud"*	***Words:*** 28.387<br>***Title:*** 1.950<br>***Abstract***: 5.015
1 + 2 + 3	Exercicio *OR Ejercicio OR "*Atividade física*" OR "Actividad física" OR "*Habilidade motora*" OR "Habilidad motora"*	***Words:*** 22.595<br>***Title:*** 3.294<br>***Abstract***: 11.320
1 + 2 + 3 + 4	*"COVID -19" OR "SARS-CoV-2" OR Coronavirus*	***Words:*** 7.705<br>***Title:*** 948<br>***Abstract***: 2.119
(Adulto) AND (Aconselhamento OR Consejo OR Conselho OR Asesoramiento OR Aconselhou OR "Promoção da saúde" OR "Promoción de la salud" OR "Educação em saúde" OR "Educación para la salud") (Exercicio OR Ejercicio OR "Atividade física" OR "Actividad física" OR "Habilidade motora" OR "Habilidad motora")AND ("COVID -19" OR "SARS-CoV-2" OR Coronavirus) [Palavras]		00

6 - Scientific Eletronic Library Online (SciELO*).* Search performed on: 03/12/2021.

Block	Descriptors	Articles
1	*Adult*	***All fields***: 21. 243 ***Abstract***: 19.158 ***Title:*** 3.461
1 + 2	*Counseling OR Counselling OR Counseled OR Counselings OR Counsellings OR Counselled OR Counsels OR "Health promotion" OR "Health education"*	***All fields***: 11.782 ***Title***: 2.283 ***Abstract:*** 9.049
1 + 2 + 3	*Exercise OR "Physical activity" OR "Motor activity"*	***All fields***: 15.239 ***Title***: 4.323 ***Abstract:*** 15.652
1 + 2 + 3 + 4	*"COVID -19" OR "SARS-CoV-2" OR Coronavirus*	***All fields***: 2.936 ***Title***: 954 ***Abstract***: 5.964
((Adult) AND (Counseling OR Counselling OR Counseled OR Counselings OR Counsellings OR Counselled OR Counsels OR "Health promotion" OR "Health education") AND (Exercise OR "Physical activity" OR "Motor activity") AND ("COVID - 19" OR "SARS-CoV-2" OR Coronavirus))		00
1	*Adulto*	***Todos los índices:*** 6.176 ***Resumen***: 4.627 ***Título:*** 1.454
1 + 2	Aconselhamento *OR Consejo OR* Conselho *OR Asesoramiento OR* Aconselhou *OR* "Promoção da saúde" *OR "Promoción de la salud" OR "*Educação em saúde" *OR "Educación para la salud"*	***Todos los índices***: 31.919 ***Resumen***: 11.402 ***Título:*** 2.822

1 + 2 + 3	Exercicio *OR Ejercicio OR* "Atividade física" *OR "Actividad física" OR* "Habilidade motora" *OR "Habilidad motora"*	***Todos los índices***: 16.108 ***Resumen***: 15.701 ***Título:*** 4.161
1 + 2 + 3 + 4	*"COVID -19" OR "SARS-CoV-2" OR Coronavirus*	***All fields***: 2.936 ***Title***: 954 ***Abstract***: 5.964
((Adulto) AND (Aconselhamento OR Consejo OR Conselho OR Asesoramiento OR Aconselhou OR "Promoção da saúde" OR "Promoción de la salud" OR "Educação em saúde" OR "Educación para la salud") AND (Exercicio OR Ejercicio OR "Atividade física" OR "Actividad física" OR "Habilidade motora" OR "Habilidad motora") AND ("COVID -19" OR "SARS-CoV-2" OR Coronavirus))		00

7 - Cumulative Index to Nursing and Allied Health Literature (CINAHL), via EBSCOhost. Search performed on: 03/12/2021.		
**Block**	**Descriptors**	**Articles**
1	*Adult*	***All fields***: 1.480.703 ***Text word***: 2.252.726
1 + 2	*Counseling OR Counselling OR Counseled OR Counselings OR Counsellings OR Counselled OR Counsels OR "Health promotion" OR "Health education"*	***All fields***: 200.523 ***Text word***: 400.444
1 + 2 + 3	*Exercise OR "Physical activity" OR "Motor activity"*	***All fields***: 263.719 ***Text word***: 405.283
1 + 2 + 3 + 4	*"COVID -19" OR "SARS-CoV-2" OR Coronavirus*	***All fields***: 40.835 ***Text word***:

		40.679
(Adult) AND (Counseling OR Counselling OR Counseled OR Counselings OR Counsellings OR Counselled OR Counsels OR "Health promotion" OR "Health education") AND (Exercise OR "Physical activity" OR "Motor activity") AND ("COVID -19" OR "SARS-CoV-2" OR Coronavirus)<br><br>***Restringir por SubjectAge:*** *Female, Male, Adult: 19-44 years, Middle Aged: 45-64 years, Aged: 65+years, Aged, 80 and over, All Adult.*		04

8 - EMBASE. Search performed on: 03/12/2021.		
**Block**	**Descriptors**	**Articles**
1	*Adult*	***All fields***: 8.955.762<br>***Text word***: 1.104.886
1 + 2	*Counseling OR Counselling OR Counseled OR Counselings OR Counsellings OR Counselled OR Counsels OR "Health promotion" OR "Health education"*	***All fields***: 540.201<br>***Text word***: 244.040
1 + 2 + 3	*Exercise OR "Physical activity" OR "Motor activity"*	***All fields***: 826.856<br>***Text word***: 543.888
1 + 2 + 3 + 4	*"COVID -19" OR "SARS-CoV-2" OR Coronavirus*	***All fields***: 230.829<br>***Text word***: 209.302

adult AND (counseling OR counselling OR counseled OR counselings OR counsellings OR counselled OR counsels OR 'health promotion' OR 'health education') AND (exercise OR 'physical activity' OR 'motor activity') AND ('covid -19' OR 'sars-cov-2' OR coronavirus) AND ([controlled clinical trial]/lim OR [randomized controlled trial]/lim) AND ([male]/lim OR [female]/lim) AND ([adult]/lim OR [young adult]/lim OR [middle aged]/lim OR [aged]/lim OR [very elderly]/lim)<br><br>*Filters applied: Age: Adult (18-64 years), Yong adult (18-24 years), Middle aged (45-64 years), Aged (65+years), Very elderly (80+years), EBM: Controlled clinical trial, randomized controlled trial, Gender: Male, female.*	23
